# The effectiveness of Kanggan granules in treating hand, foot and mouth disease in children: a meta-analysis

**DOI:** 10.3389/fped.2026.1890367

**Published:** 2026-09-08

**Authors:** Zhi Zhu

**Affiliations:** Pharmacy Department, 903 Hospital, Jiangyou, China

**Keywords:** hand, foot and mouth disease, Kanggan granules, meta-analysis, systematic review, traditional Chinese medicine

## Abstract

**Background:**

Hand, foot, and mouth disease (HFMD) is a common infectious disease in children caused by enteroviruses, and effective specific antiviral therapies remain limited. Kanggan granules, a traditional Chinese medicine formulation, have been widely used as an adjunctive treatment for HFMD. This study aimed to systematically evaluate the clinical efficacy and safety of Kanggan granules in children with HFMD.

**Methods:**

A systematic literature search was conducted in PubMed, Embase, Cochrane Library, CNKI, Wanfang Data, and VIP databases from inception to June 30, 2024. Randomized controlled trials (RCTs) comparing Kanggan granules with or without conventional treatment vs. conventional treatment alone were included. Two reviewers independently performed study selection, data extraction, and risk-of-bias assessment using the Cochrane RoB 2 tool. Meta-analyses were conducted using random-effects or fixed-effects models according to heterogeneity. The certainty of evidence was evaluated using the GRADE framework.

**Results:**

Thirteen RCTs involving 1,320 children with HFMD were included. Compared with conventional treatment alone, Kanggan granules significantly increased the total effectiveness rate (OR = 4.60, 95% CI: 2.86–7.43, *p* < 0.001). Kanggan granules-based treatment was also associated with shorter herpes regression time (MD = −1.41 days, 95% CI: −1.81 to −1.00), oral ulcer healing time (MD = −1.08 days, 95% CI: −1.42 to −0.74), fever duration (MD = −1.32 days, 95% CI: −1.58 to −1.05), and hospitalization duration (MD = −1.90 days, 95% CI: −2.62 to −1.19). However, substantial heterogeneity was observed for several continuous outcomes. Safety data were insufficient, with only one study reporting adverse events.

**Conclusions:**

Kanggan granules may provide potential clinical benefits for children with HFMD as an adjunctive therapy. However, the certainty of evidence remains limited due to methodological weaknesses, substantial heterogeneity, possible publication bias, and insufficient safety reporting. Further high-quality, multicenter, double-blind RCTs with standardized outcomes and comprehensive safety monitoring are required.

## Introduction

Hand, foot, and mouth disease (HFMD) is an infectious disease commonly diagnosed in children caused mainly by over 20 enterovirus types, especially among children 5 years of age and younger. The classic manifestations of HFMD include oral ulceration, anorexia, low-grade fever, and small vesicles or ulcers on the hands, feet, and mouth (1). HFMD is a very contagious disease that quickly spreads and is capable of causing outbreaks in crowded circumstances such as schools (2, 3). While most children recover in a week, specific HFMD infections can cause severe complications, including viral myocarditis, pulmonary edema, aseptic meningoencephalitis, and neuroinflammatory cognitive impairment. Additionally, a small subset of severely ill children can experience rapid deterioration and die (4, 5). As a result, the importance of early prevention and timely intervention in understanding HFMD cannot be overstated. Western medicine frames contexts involving the disease primarily with symptomatic treatment, enterovirus containment, prevention of secondary bacterial infection, and symptomatic treatment. Broad-spectrum antiviral and anti-infective drugs are often prescribed to children with HFMD, as there is no specific drug to treat enterovirus-associated diseases. Unfortunately, the use of broad-spectrum medications results in consequences such as drug-resistant pathogens and adverse events (6).

As part of complementary and alternative medicine (CAM), Traditional Chinese Medicine (TCM) has heretofore effectively demonstrated considerable benefits in an array of infectious disease antiviral treatments. Additionally, extensive clinical practice has illustrated both efficacy and safety in the use of various Chinese patent medicine oral liquids derived from natural herbs, commonly in treating viral HFMD in children. Not only did these liquid medications have clinical evidence demonstrating their clinical antiviral effectiveness, but they also substantially increased children's immune responses (7). One example of a patent Chinese medicine is “Kanggan” granules, which are commonly administered to children with HFMD. The primary constituents of Kanggan granules include Lonicera japonica Flos (honeysuckle flower), Paeonia lactiflora Radix (red peony root), and Corydalis yanhusuo Rhizoma (Corydalis rhizome). Even modern pharmacological studies indicate that the Kanggan granules formulation inhibits enteroviruses, has anti-inflammatory effects, and promotes tissue healing (8).

Moreover, Kanggan granules have been reported to regulate inflammatory responses and immune-related pathways. Previous pharmacological studies have suggested that Kanggan granules may possess anti-inflammatory and antiviral properties; however, these mechanisms were not directly evaluated in the included clinical trials.

Clinical studies also demonstrate that Kanggan granules were meaningfully effective for treating HFMD, and the associated symptoms of fever, herpes, and oral ulcers. The clinical experience has led some practitioners to conclude a meaningful additive effect of Kanggan granules to conventional treatment methods. Even with promising evidence, there has been significant ongoing debate on the effective methods and specific efficacy of the Chinese patent medicine Kanggan granules to treat HFMD and the entirety of associated symptoms. The debate largely evinces some of the concerns related to sample size, flawed trials, clinical trial reporting, and lack of long-term follow-up. This proposed study, therefore, aims to conduct a systematic review with meta-analysis of clinical outcomes relating to the efficacy of Kanggan granules to treat HFMD, and to provide more sound reference material for clinicians' choice of treatment.

## Methods

### Literature search

A comprehensive search of both English and Chinese databases, including PubMed, Embase, the Cochrane Library, China National Knowledge Infrastructure (CNKI), Wanfang Data, and VIP, was conducted from database inception to June 30, 2024. The protocol of this systematic review was prospectively registered in the International Platform of Registered Systematic Review and Meta-analysis Protocols (INPLASY) (registration number: INPLASY202670115). The Chinese and English search strategies, eligibility criteria, and analytical methods were predefined according to the registered protocol.

The search strategy combined intervention-related terms and disease-related terms using Boolean operators. The English search strategy was: (“Kanggan granules” OR “Kan-gan granules” OR “Kanggan granules”) AND (“hand-foot-mouth disease” OR “hand, foot, and mouth disease” OR “HFMD”). The Chinese search strategy included equivalent Chinese terms for Kanggan granules and hand, foot, and mouth disease. The complete search strategies for PubMed and CNKI are provided in Supplementary Appendix S1.

No language restrictions were applied during the literature search. Grey literature, conference proceedings, dissertations, and clinical trial registration platforms, including ClinicalTrials.gov and the Chinese Clinical Trial Registry (ChiCTR), were not searched because this review focused on published randomized controlled trials. The screening process was summarized in a PRISMA flowchart, describing the number of identified, screened, excluded, and included studies.

**Figure 1:**
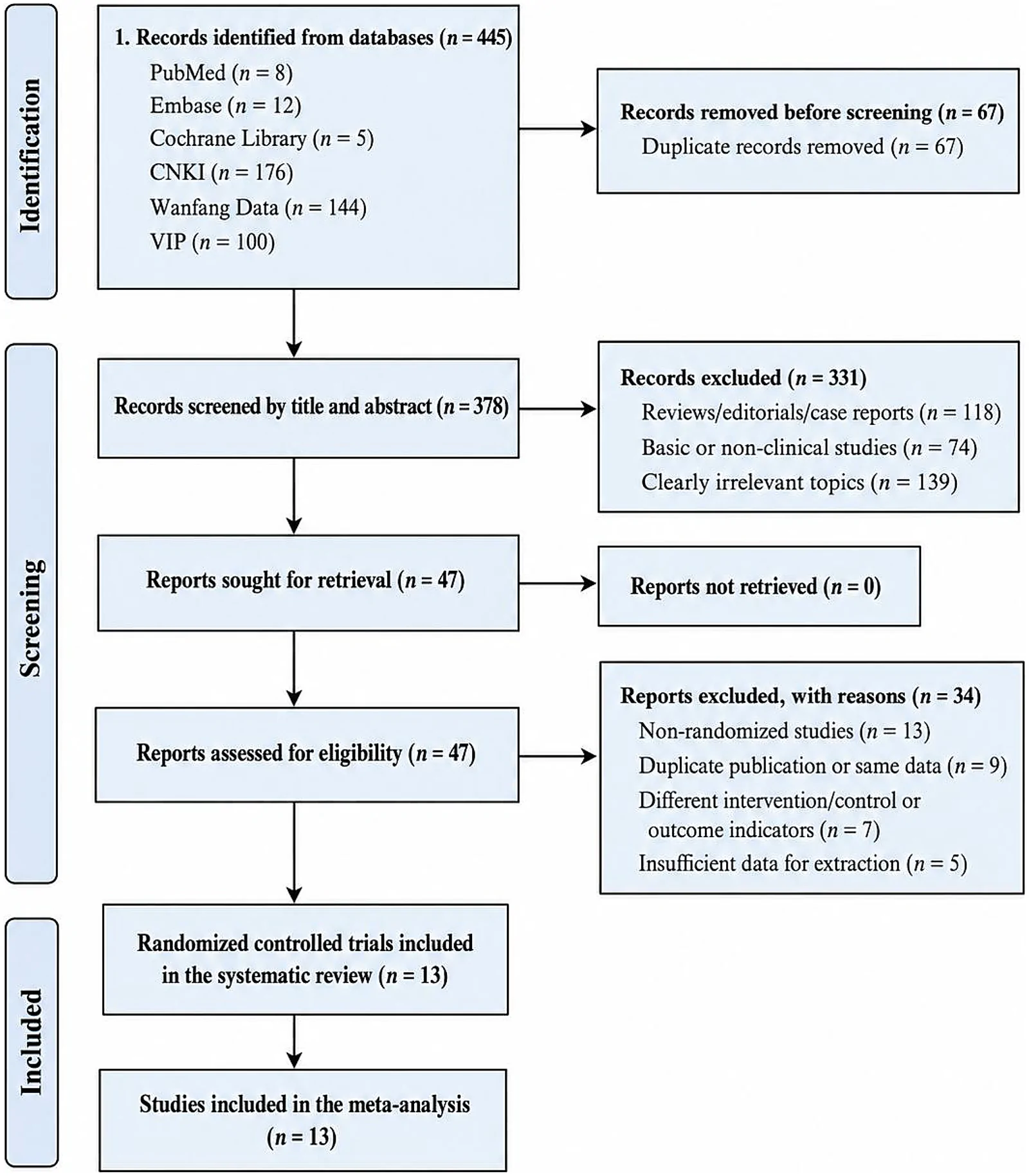
PRISMA flow diagram of the study selection process. This figure illustrates the process of literature identification, screening, eligibility assessment, and final inclusion of randomized controlled trials in accordance with PRISMA guidelines.

Additionally, subject terms and keywords were searched using “Kanggan granules” as the primary search term. The search period spanned from the establishment of each database to June 30, 2024. The screening process was summarized in a flowchart according to PRISMA guidelines, which also describes the number of records identified, screened, excluded, and ultimately included in the meta-analysis (Figure 1).

### Inclusion and exclusion criteria

Randomized controlled trials (RCTs) comparing Kanggan granules with or without conventional drugs (experimental group) vs. conventional drugs alone (control group) for the treatment of HFMD were included in the study, regardless of blinding. The inclusion criteria were as follows: 1) any study involving patients meeting the diagnostic criteria for HFMD (9–11), regardless of age or race; 2) kanggan granules, with or without conventional drugs, were administered to the experimental group as the intervention; 3) conventional drug treatment excluding Kanggan granules (ribavirin, Shuanghuanglian, Yanhuning, recombinant human interferon *α*-1b, and other drugs) was administered to the control group; 4) studies evaluated one of more of the outcome measures including, clinical effective, time to regression of herpes, time for resolution of oral ulcers, time for fever reduction, and length of stay; 5) all clinical studies were RCTs.

The exclusion criteria were: 1) non-RCT studies, such as reviews, basic research, case reports, systematic evaluations, and meta-analyses; 2) Literature with duplication of publications or the same data; 3) Literature with completely different intervention measures or outcome indicators. Two reviewers independently performed study selection and data extraction. Any disagreement was resolved through discussion or consultation with a third reviewer. Any disagreement during study selection or data extraction was resolved through discussion or consultation with a third reviewer.

### Data extraction and quality assessment

The data, which was independently collected by two reviewers, included the first author, year of publication, sample size, intervention, duration of treatment, baseline characteristics, and outcome indicators. The criteria used to evaluate clinical effectiveness were extracted from each included study. In general, the clinical response was categorized as markedly effective, effective, or ineffective. Markedly effective was defined as normalization of body temperature, disappearance of oral herpes/ulcers, and healing of ulcer surfaces after treatment. Effective was defined as partial improvement of symptoms, including reduced fever, partial resolution of oral lesions, or decreased ulcer size. Ineffective was defined as persistent symptoms without obvious improvement or disease progression. The total effective rate was calculated as the proportion of patients classified as markedly effective or effective among all treated patients. Because the specific definitions varied slightly among the included trials, total effective rate was considered a composite clinical outcome and interpreted cautiously. Other indicators included ② duration of herpes disappearance, ③ duration of oral ulcer healing, ④ duration of fever reduction, and ⑤ duration of hospitalization.

Risk of bias was assessed independently by two reviewers using the Cochrane Risk of Bias 2 (RoB 2) tool for randomized controlled trials. The assessment covered five domains: (1) bias arising from the randomization process, (2) bias due to deviations from intended interventions, (3) bias due to missing outcome data, (4) bias in measurement of the outcome, and (5) bias in selection of the reported result. Each domain was classified as “low risk of bias”, “some concerns”, or “high risk of bias”. Disagreements were resolved through discussion with a third reviewer. The overall risk-of-bias assessment was presented using RoB 2 traffic-light plots and summary figures. While some studies were assessed as having a generally low risk of bias for some domains, their overall quality was affected by unclear allocation concealment, inconsistent blinding, and inconsistently published protocol, and should be kept in mind when interpreting the results of our findings. A third-party reviewer verified all the data extraction and risk of bias assessment data to enhance the reliability of the review.

### Statistical methods

The meta-analysis was conducted via RevMan 5.3 software. Dichotomous variables are represented as odds ratios (ORs) with 95% confidence intervals (CIs), and continuous variables are expressed as the mean difference (MD) with 95% CI. If *P* > 0.1 and I^2^ ≤ 50%, no statistical heterogeneity was observed among the studies, and the fixed-effects model was used for analysis. Otherwise, the random-effects model was employed. Sensitivity analysis was conducted via the leave-one-out method to assess the impact of individual studies on the overall combined results. Sources of heterogeneity were further explored through subgroup analyses according to intervention characteristics, control treatment regimens, treatment duration, disease severity, sample size, and methodological quality when sufficient data were available. Meta-regression was not performed because the number of included studies for individual outcomes was insufficient to provide reliable estimates. Potential publication bias was assessed using funnel plots when sufficient studies were available. Funnel plot interpretation was restricted to outcomes with ≥10 studies because analyses involving fewer studies have limited reliability.

The certainty of evidence for the main outcomes was assessed using the Grading of Recommendations Assessment, Development and Evaluation (GRADE) framework. The certainty of evidence was rated as high, moderate, low, or very low based on risk of bias, inconsistency, indirectness, imprecision, and publication bias. A Summary of Findings (SoF) table was generated for the primary outcomes, including total effectiveness rate, fever reduction time, herpes regression time, oral ulcer healing time, and hospitalization duration.

## Results

### Literature screening results

A total of 445 studies in Chinese-language were screened. Titles and abstracts were assessed and duplicates, reviews and non-clinical studies were removed, leaving 13 RCTs that met inclusion (12–24). 10 studies compared Kanggan granules along with standard medicine vs. standard medicine, and there were 3 studies that compared Kanggan granules vs. standard medicine. The process for identifying, screening, eligibility, and final inclusion of studies is in the PRISMA flowchart (Figure 1). Details of included studies are included in Table 1.

**Table 1:** Basic characteristics of included studies .

Study	Sample size		Age^b^		Proportion of severe cases	Diagnostic criteria	Interventions		Treatment period	Outcomes^a^
	Experimental group	Control group	Experimental group	Control group			Experimental group	Control group		
Zhou Hong (13)	63	63	3.62 ± 1.12	3.57 ± 1.21	Not report	Hand, Foot, and Mouth Disease (HFMD) Diagnosis and Treatment Guidelines (2010 Edition)	Kanggan Granules + Ribavirin + Yanhuning	Ribavirin + Yanhuning	3–5 days	①②③④⑤
Ji Jing (14)	68	68	2.8 (0.67–6)	2.6 (0.58–6)	1.5%	Not report	Kanggan Granules + Yanhuning	Yanhuning	3–5 days	①②③④⑤
Li Yuyu (15)	43	43	3.5 ± 1.5	3.4 ± 1.3	Not report	Not report	Kanggan Granules + Shuanghuanglian	Shuanghuanglian	Not report	①
Qiu jing (16)	58	57	2.38 ± 0.8	2.41 ± 0.8	Not report	Not report	Kanggan Granules + Ribavirin + Yanhuning	Ribavirin + Yanhuning	5 days	①
Yang Jianwen (17)	163	180	2.82 ± 3.56	Not report	Enterovirus (EV71) Infection Diagnosis and Treatment Guidelines (2008 Edition)	Kanggan Granules + Ribavirin	Ribavirin	Not report	①②③④	
Li Jun (18)	50	46	Median: 1.3	Median:1.5	0%	7th Edition of Zhu Futang Practical Pediatrics	Kanggan Granules + Ribavirin	Ribavirin	Not report	②④⑤
Wang Ping (19)	30	30	3.93 ± 1.29	3.79 ± 1.47	0%	Not report	Kanggan Granules + Ribavirin	Ribavirin	Not report	②③④⑤
Zhao Huaqing (20)	61	61	3.33 ± 0.32	3.28 ± 0.25	0%	Hand, Foot, and Mouth Disease (HFMD) Diagnosis and Treatment Guidelines (2010 Edition)	Kanggan Granules + Ribavirin	Ribavirin	5–7 days	①②③④
Zheng Min (21)	75	75	3.02 ± 1.18	2.83 ± 1.04	Not report	Not report	Kanggan Granules + Ribavirin	Recombinant human interferon *α*-1b	5 days	①②③④⑤
Zhang Guizhou (22)	38	38	5.28 ± 0.65	5.19 ± 0.68	5.3%	Hand, Foot, and Mouth Disease (HFMD) Diagnosis and Treatment Guidelines (2018 Edition)	Kanggan Granules + Ribavirin	Ribavirin	10 days	①②③④
Kang Bi (23)	18	8	NA	Not report	Not report	Kanggan Granules	Ribavirin	Not report	①	
Zhang Shuping (24)	30	30	3.10 ± 1.2	3.13 ± 1.21	Not report	Not report	Kanggan Granules	Shuanghuanglian	Not report	①
Zhu Baogang (25)	50	30	3.68 ± 1.21	Not report	Not report	Kanggan Granules	Shuanghuanglian	Not report	①	

a Outcomes: ① Total effective rate, ② time for herpes disappearance, ③ time for oral ulcer healing, ④ time for fever reduction, and ⑤ duration of hospitalization.

b Mean ± Standard deviation; Median (Minium—Maximum).

### Methodological quality evaluation

**Figure 2:**
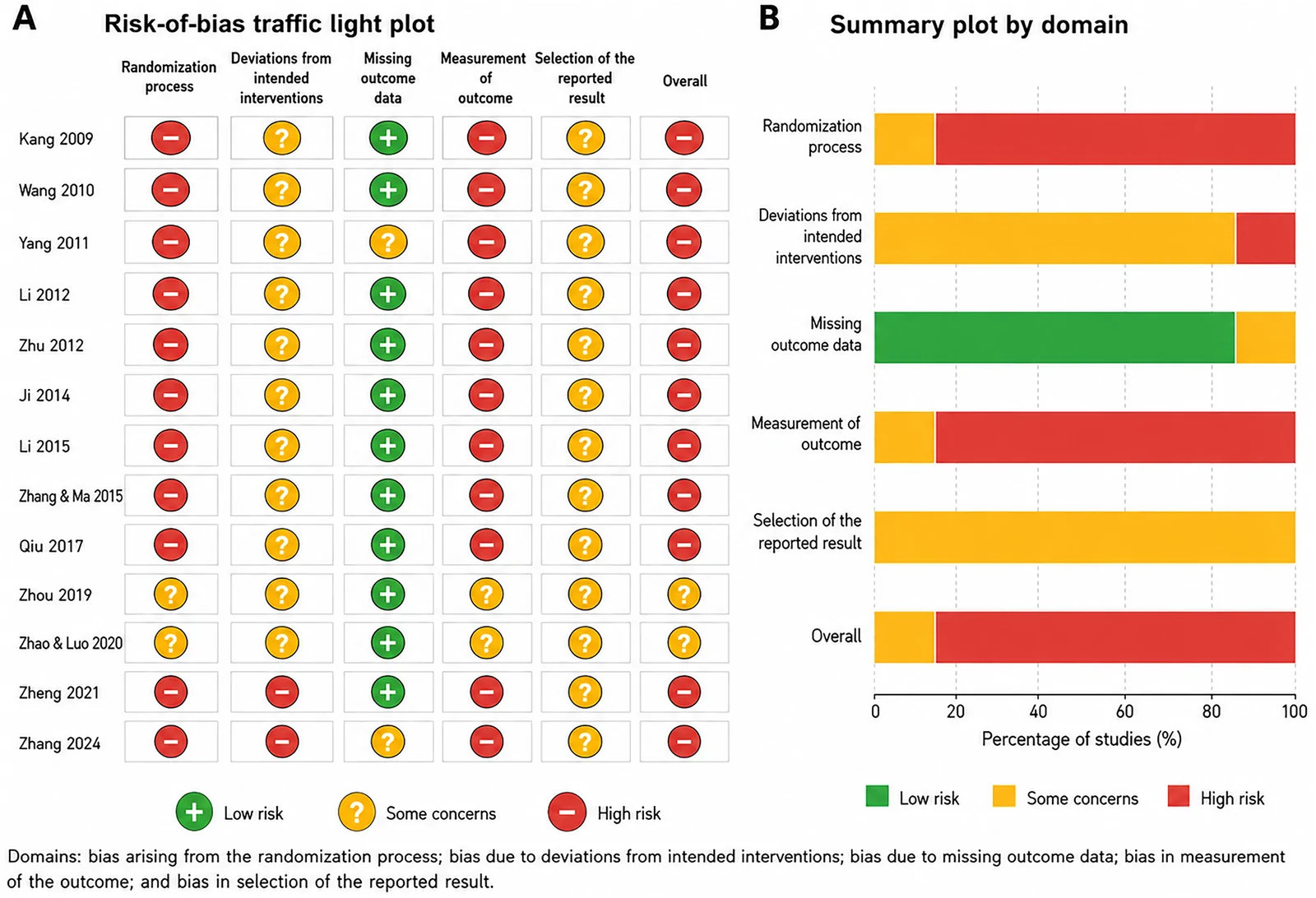
Risk-of-bias assessment of included randomized controlled trials using the cochrane RoB 2 tool. **(A)** Risk-of-bias traffic light plot. **(B)** Summary plot by domain.

All included studies were randomized controlled trials. According to the RoB 2 assessment, concerns were mainly identified in the domains of randomization process and measurement of outcomes. Although several studies reported random allocation, details regarding allocation concealment were insufficient. Furthermore, most trials did not report blinding of participants, investigators, or outcome assessors, resulting in potential concerns regarding deviations from intended interventions and outcome measurement bias. These methodological limitations may have reduced confidence in the pooled estimates and were considered in the subsequent GRADE assessment of evidence certainty. Further details are described in Figure 2.

### Subgroup classification

Due to variability in the interventions and clinical characteristics across the 13 RCTs, subgroup analyses were performed according to treatment regimen, control intervention, and methodological quality. Additional subgroup comparisons based on treatment duration and study characteristics were considered where sufficient information was available. These analyses were performed to explore potential sources of heterogeneity among the pooled outcomes (Table 2).

**Table 2:** Subgroup analyses of clinical outcomes according to intervention strategy and study characteristics.

Outcomes	Subgroup characteristics	No. of studies	I^2^ (%)	OR/MD	95% CI	*p* value
Total effectiveness rate	Kanggan plus conventional treatment vs. conventional treatment	8	0%	4.6	2.86, 7.43	<0.001
	Kanggan monotherapy vs. conventional treatment	3	0%	7.93	2.19, 28.78	0.003
	Moderate risk of bias	2	0%	4.72	1.86, 12.00	0.001
	High risk of bias	9	0%	5	3.00, 8.32	<0.001
Herpes regression time	Kanggan plus conventional treatment vs. conventional treatment	6	95.20%	−1.27	−1.71, −0.82	<0.001
	Kanggan monotherapy vs. conventional treatment	2	98.20%	−1.84	−3.54, −0.13	0.035
	Moderate risk of bias	2	98.10%	−1.41	−2.45, −0.37	0.008
	High risk of bias	6	94.30%	−1.39	−2.03, −0.74	<0.001
Oral ulcer healing time	Kanggan plus conventional treatment vs. conventional treatment	6	95.70%	−1.04	−1.57, −0.51	<0.001
	Moderate risk of bias	2	97.90%	−1.26	−2.26, −0.25	0.015
	High risk of bias	5	90.70%	−0.99	−1.54, −0.44	<0.001
Fever duration	Kanggan plus conventional treatment vs. conventional treatment	6	96.20%	−1.31	−1.64, −0.98	<0.001
	Kanggan monotherapy vs. conventional treatment	2	74.70%	−1.36	−1.81, −0.90	<0.001
	Moderate risk of bias	2	0%	−1.22	−1.30, −1.14	<0.001
	High risk of bias	6	96.40%	−1.35	−1.71, −1.00	<0.001
Hospital stay	Kanggan plus conventional treatment vs. conventional treatment	3	88.60%	−1.83	−2.48, −1.19	<0.001
	Kanggan monotherapy vs. conventional treatment	2	98.30%	−1.96	−3.93, 0.01	0.051
	High risk of bias	4	96.40%	−1.85	−2.75, −0.94	<0.001

CI, Confidence Interval; MD, Mean Difference; OR, Odds Ratio.

### Meta-analysis results

#### Total effective rate

**Figure 3:**
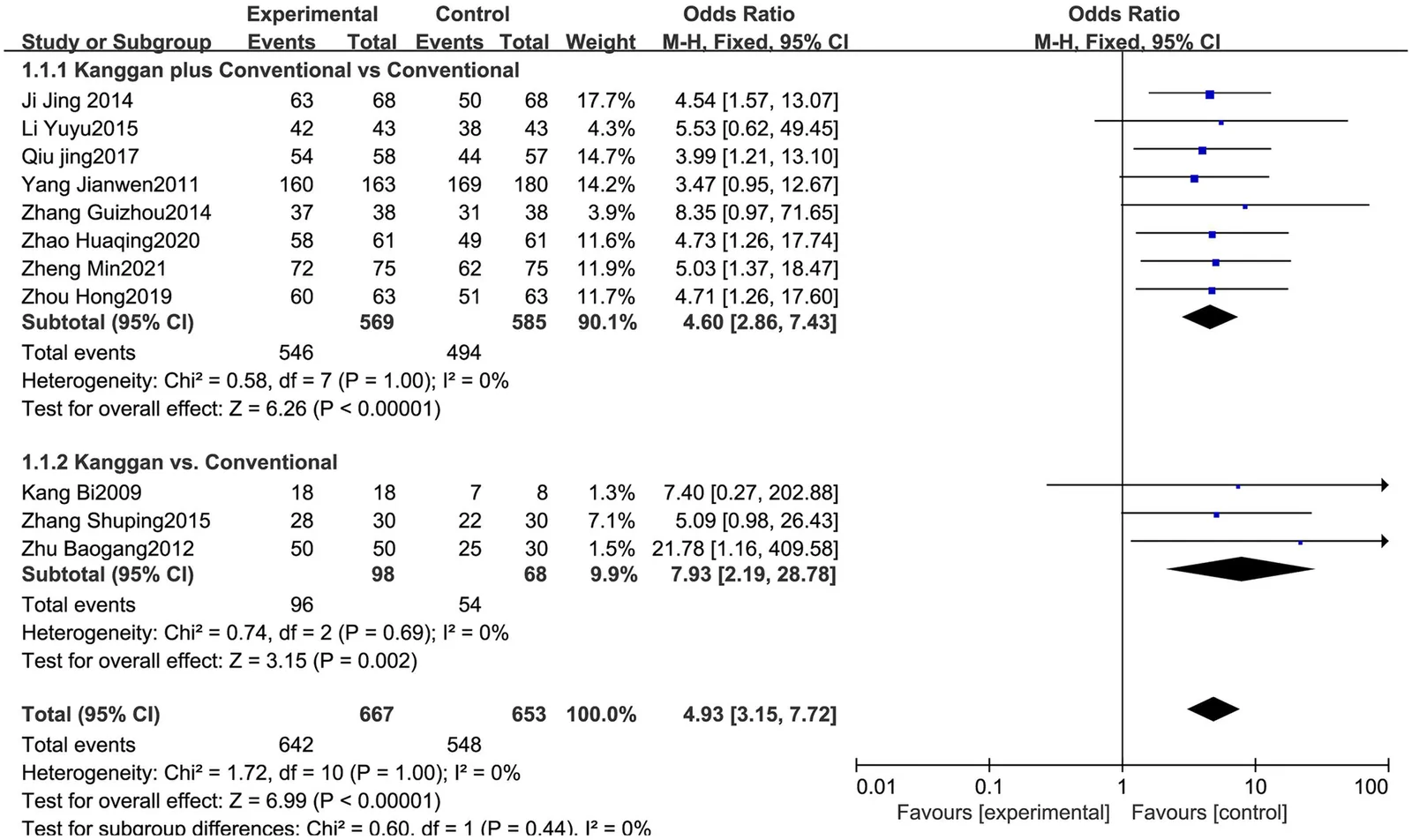
Forest plot of the effect of Kanggan granules on total effectiveness rate in children with hand, foot, and mouth disease. Forest plot showing the pooled odds ratio for total effectiveness rate between Kanggan granules-based treatment and control groups. The pooled effect was calculated using a Mantel–Haenszel fixed-effects model. Squares represent individual study estimates, horizontal lines indicate 95% confidence intervals, and diamonds represent pooled estimates.

Eleven studies reported total effective rates and included 667 patients in the experimental group and 653 patients in the control group. No significant heterogeneity was observed among these studies (*P* > 0.1); therefore, a fixed-effects model was applied. The pooled analysis showed that Kanggan granules alone or combined with conventional treatment were associated with a higher total effective rate compared with conventional treatment alone (OR = 4.60, 95% CI 2.86–7.43, *p* < 0.001). However, because total effective rate represents a composite outcome combining symptom improvement categories and may vary slightly among studies, these findings should be interpreted together with more objective clinical outcomes, including fever reduction time, herpes regression time, and hospitalization duration. In subgroup analysis, Kanggan granules monotherapy also showed a favorable effect compared with conventional treatment alone (I^2^ = 0%, OR = 7.93, 95% CI 2.19–28.78, *p* < 0.001). When stratified by risk of bias, studies classified as having moderate risk of bias showed a slightly smaller pooled effect estimate (OR = 4.72, 95% CI 1.86–12.00, *p* = 0.001) compared with studies at high risk of bias, although the limited number of studies precluded definitive conclusions regarding the influence of methodological quality (Table 2). Outcomes are presented in Figure 3.

##### Herpes regression time

**Figure 4:**
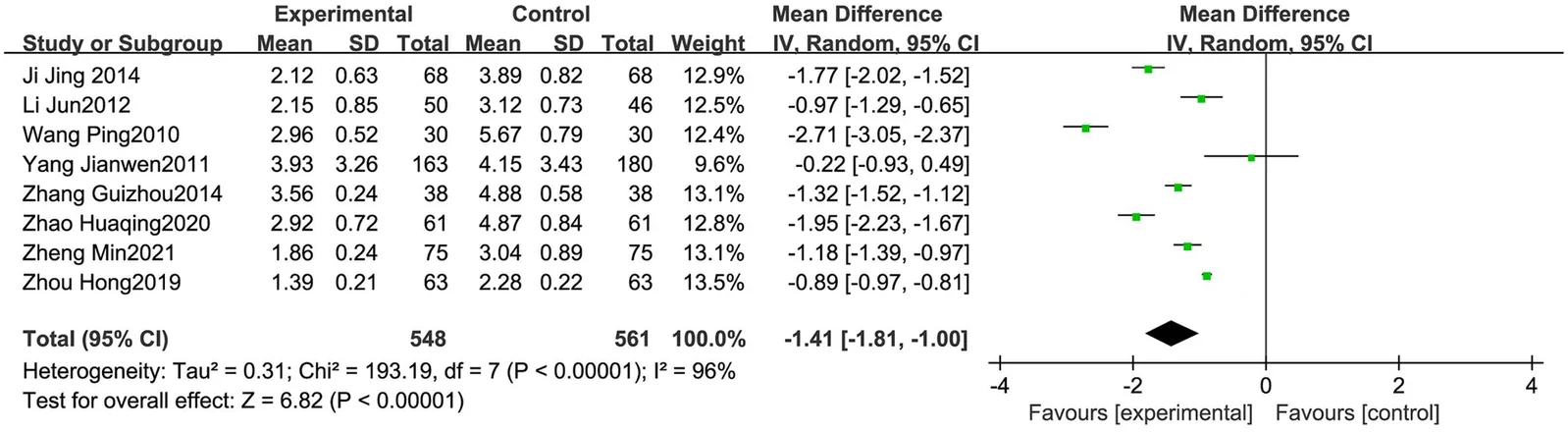
Forest plot of the effect of Kanggan granules on herpes regression time in children with hand, foot, and mouth disease. Forest plot showing the pooled mean difference in herpes regression time between Kanggan granules-based treatment and control groups. The analysis was performed using a random-effects model. Squares represent individual study estimates, horizontal lines indicate 95% confidence intervals, and the diamond represents the pooled effect estimate.

Eight studies involving 548 experimental participants and 561 control participants reported herpes regression time. The random-effects model was utilized because of significant heterogeneity. MD was −1.41 (95% CI −1.81 to −1.00, *p* < 0.001, I^2^ = 96%), favoring Kanggan granules plus conventional agents. Subgroup analyses indicated that variations in background conventional treatments and methodological quality among included studies may partially explain the observed heterogeneity. (Table 2). The pooled results are presented in Figure 4.

##### Oral ulcer healing time

**Figure 5:**
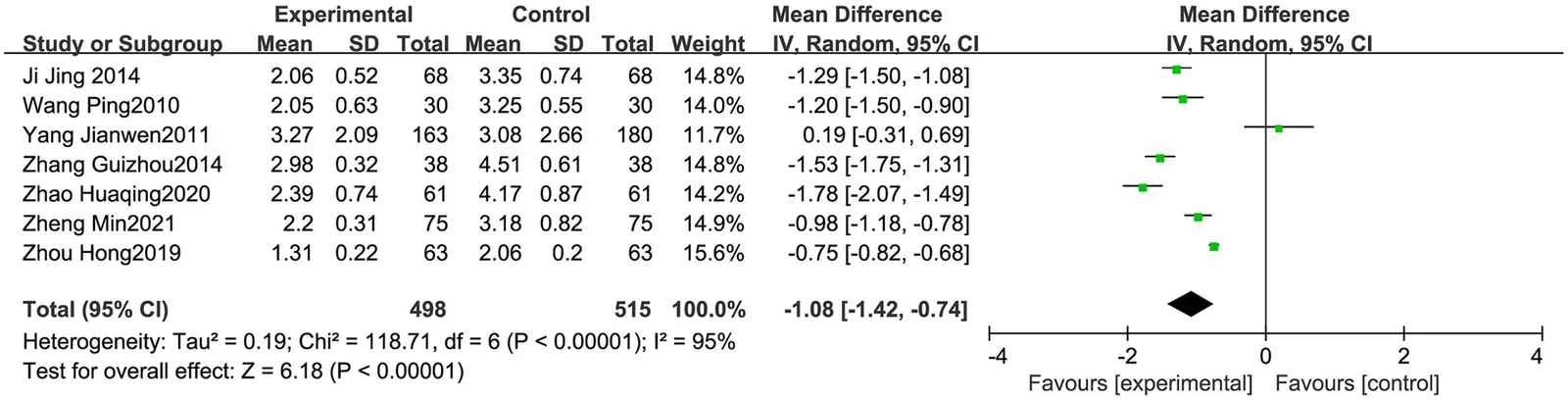
Forest plot of the effect of Kanggan granules on oral ulcer healing time in children with hand, foot, and mouth disease. Forest plot showing the pooled mean difference in oral ulcer healing time between Kanggan granules-based treatment and control groups. The pooled effect was calculated using a random-effects model. Squares represent individual study estimates, horizontal lines indicate 95% confidence intervals, and the diamond represents the overall pooled effect.

Seven trials (498 experimental, 515 control) investigated oral ulcer healing time. The mean difference (MD) was −1.08 (95% CI −1.42 to −0.74, *p* < 0.001, 95%) using a random-effects model (Figure 5).

##### Fever reduction time

**Figure 6:**
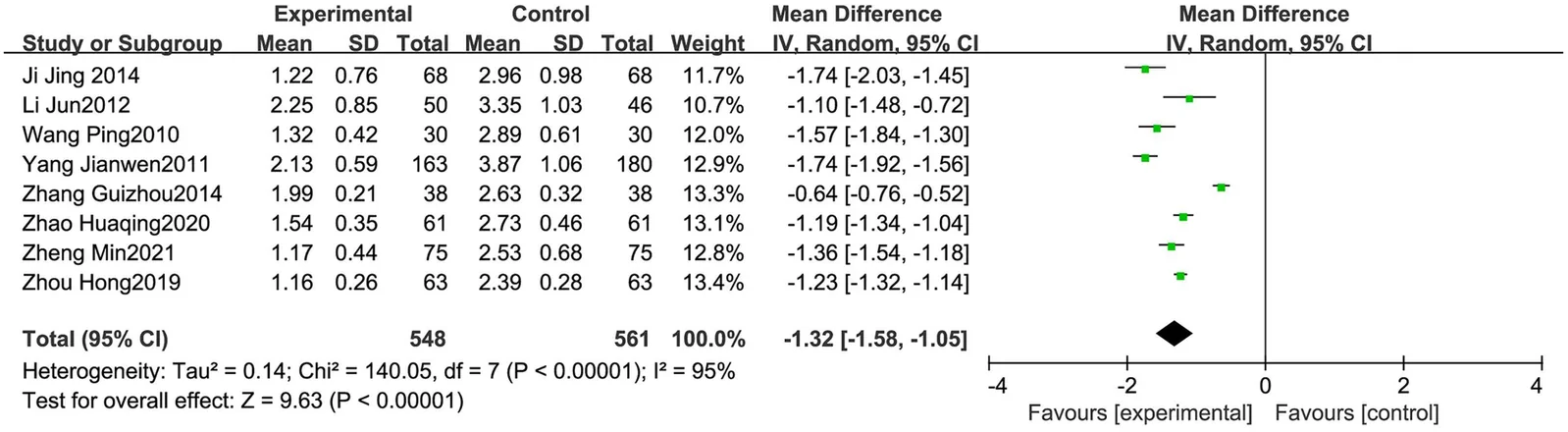
Forest plot of the effect of Kanggan granules on fever duration in children with hand, foot, and mouth disease. Forest plot showing the pooled mean difference in fever duration between Kanggan granules-based treatment and control groups. The pooled estimate was calculated using a random-effects model. Squares represent individual study effects, horizontal lines indicate 95% confidence intervals, and the diamond represents the overall pooled effect.

Eight studies involved 548 experimental patients and 561 control patients. The MD was −1.32 (95% CI −1.58 to −1.05, *p* < 0.001, I^2^ = 95%). Given that subgroup results indicated a consistent beneficial effect, differences in methodological rigor, conventional treatment regimens, and treatment duration may have contributed to the substantial heterogeneity observed among studies (Table 2). The results are presented in Figure 6.

##### Hospitalization time

Five studies reported the length of hospitalization with 286 participants in the experimental group and 282 in the control group. The mean difference was −1.90 (95% CI −2.62 to −1.19, *p* < 0.001, I^2^ = 96%) in favor of Kanggan plus conventional treatment (Supplementary Figure S1). As with other results, differences in study design, baseline treatment protocols, disease severity, and sample size may have contributed to the observed heterogeneity (Table 2).

##### Sensitivity analysis

A sensitivity analysis limited to “high-quality” trials was unable to be performed because there was low methodological quality. Leave-one-out analysis showed that no one individual study significantly impacted the pooled results. The overall confidence was very limited due to inconsistent reporting of allocation concealment and blinding (Supplementary Figure S2).

##### Certainty of evidence assessment

The certainty of evidence was assessed using the GRADE framework (Supplementary Table S1). The certainty of evidence for the primary outcomes ranged from low to very low, mainly due to concerns regarding risk of bias, inconsistency, and imprecision. Although Kanggan granules showed favorable effects on total effectiveness rate and symptom resolution outcomes, these findings should be interpreted cautiously because most included trials lacked adequate allocation concealment and blinding.

##### Publication bias assessment

The funnel plot, which assessed potential publication bias for total effective rates (Supplementary Figure S3), was asymmetrical (*p* = 0.007), indicating the possibility that small, negative trials are missing, leading to a possible overestimate of the observed benefits. After imputing the potentially missing studies by trim and fill method, the pooled results still demonstrated that Kanggan Granules were significantly more effective than the control group in terms of total effective rate (OR: 4.43; 95% CI: 2.90–6.77; *p* < 0.001). The forest plot and funnel plot after trim-and-fill correction are presented in Supplementary Figure S4.

##### Safety outcomes

Safety data were insufficiently reported among the included studies. Only one trial (Zhao and Luo, 2020) reported adverse events, including nausea, diarrhea, and vomiting, with no significant difference between the Kanggan granules group and the control group. The remaining 12 studies did not provide detailed safety information or systematic adverse-event monitoring. Therefore, the safety profile of Kanggan granules could not be comprehensively evaluated (Supplementary Table S1).

## Discussion

The composition of Kanggan granules includes three traditional Chinese medicinal herbs: *Lonicera japonica* Flos, *Paeonia lactiflora* Radix, and *Corydalis yanhusuo* Rhizoma. Among them, honeysuckle and red peony root are recognized for their heat-clearing and detoxifying properties, while red peony root additionally clears heat, cools the blood, prevents blood stasis, and alleviates pain. Modern pharmacological studies have confirmed that decoctions containing these herbs exhibit inhibitory effects against influenza virus, parainfluenza virus, enterovirus, herpesvirus, and other pathogens (25). This study systematically evaluated the clinical efficacy of Kanggan granules in treating HFMD, focusing on parameters such as total effective rate, herpes regression time, ulcer healing time, fever reduction time, and hospitalization duration. Meta-analysis results demonstrated that Kanggan granules, whether used alone or combined with conventional treatment, significantly improved the total effective rate and shortened the times to symptom resolution and hospitalization compared with conventional treatment alone.

Consistent with prior studies on heat-clearing and detoxifying Chinese patent medicines, our findings support the adjunctive role of such formulations in mild HFMD. Shiyan et al. reported that the addition of heat-clearing and detoxifying Chinese medicine to conventional therapy effectively slowed disease progression and accelerated mucosal healing, although differences in drug types across studies may have contributed to heterogeneity (26). Similarly, a meta-analysis by Wen-Yi et al. demonstrated that Xiaoer Chaigui Tuire Granule significantly shortened oral ulcer healing time and fever duration, with no serious adverse reactions observed (27). These observations are broadly consistent with our results, reinforcing the potential value of TCM adjunctive therapy while highlighting the need for standardized interventions in future research.

The observed clinical benefits of Kanggan granules may be related to their potential anti-inflammatory and immunomodulatory properties; however, the underlying mechanisms remain uncertain because mechanistic outcomes were not directly assessed in the included clinical trials. Song et al. employed network pharmacology and molecular docking methods and suggested that Kanggan granules might exert biological effects through the PI3K-AKT signaling pathway, thereby inhibiting inflammatory responses and regulating immune function (28). At the clinical biomarker level, Hu et al. confirmed that Kanggan granules effectively reduced peripheral inflammatory factors in children with HFMD and promoted increases in immune cell counts (29). Furthermore, Li et al. reported that the drug significantly reduced the levels of IL-2, IL-4, IL-6, IL-10, TNF-*α*, and IFN-*γ*, while increasing CD3⁺, CD4⁺, and CD8⁺ immune cells (30). These biomarker changes are biologically plausible, as HFMD pathogenesis is known to involve dysregulated inflammatory and immune responses. Zeng et al. found that inflammatory cytokines including IL-3, IL-6, IL-12, and TNF-*α* were significantly elevated in HFMD patients, suggesting a systemic inflammatory response that may contribute to severe complications (31). Qianwen et al. further reported altered Th1/Th2 cell balance and cytokine profiles in EV71-associated HFMD, with platelets from mild cases promoting Th1 differentiation and IFN-*γ* secretion (32). Taken together, the available biomarker evidence provides a plausible biological framework, but it is critical to emphasize that none of these mechanistic pathways were directly evaluated within the included RCTs. Therefore, all mechanistic interpretations remain speculative and hypothesis-generating, requiring rigorous confirmation in future dedicated studies.

Several important limitations of this meta-analysis must be acknowledged. Although the protocol was prospectively registered in INPLASY, we did not register in PROSPERO because this review focused on a specific TCM intervention; nevertheless, predefined eligibility criteria were applied to minimize reporting bias. First, the overall certainty of evidence, assessed using the GRADE framework, was rated as low to very low for all major outcomes, primarily due to methodological weaknesses identified by the RoB 2 assessment—most trials lacked adequate allocation concealment, blinding, and clear outcome measurement protocols. Second, substantial statistical heterogeneity (I^2^ > 95%) was observed for continuous outcomes. The control groups varied considerably in their conventional treatments, including ribavirin, interferon-based therapies, Shuanghuanglian, Yanhuning, and supportive symptomatic treatments, which—along with differences in disease severity, treatment duration, sample size, and methodological quality—may have contributed to the high heterogeneity. Although subgroup analyses were performed, the limited number of studies precluded reliable meta-regression. Third, incomplete reporting of withdrawals and missing data, together with the geographic restriction to studies conducted solely in China, limits the generalizability of our findings. Fourth, safety evidence remains insufficient; only one trial (33) reported adverse events (nausea, diarrhea, and vomiting), with no significant intergroup difference, whereas the remaining 12 studies provided no systematic safety monitoring. Fifth, publication bias cannot be excluded, as suggested by funnel plot asymmetry; moreover, grey literature, conference abstracts, dissertations, and trial registries were not searched, which may have overestimated the pooled effects. Finally, the reliance on “total effective rate”—a subjective composite outcome with variable definitions across studies—may have introduced measurement bias; hence, greater emphasis should be placed on more objective endpoints such as fever duration, ulcer healing, and hospitalization.

## Conclusions

This meta-analysis suggests that Kanggan granules, alone or as an adjunct to conventional therapy, may offer potential clinical benefits for children with HFMD by improving symptom resolution and shortening recovery time. However, owing to the low-to-very-low certainty of evidence, substantial heterogeneity, potential publication bias, and insufficient safety data, these findings should be interpreted with considerable caution. Well-designed, multicenter, double-blind, prospectively registered randomized controlled trials with standardized outcome definitions, homogeneous background therapies, and comprehensive adverse-event monitoring are urgently needed to confirm the efficacy and safety of Kanggan granules before any strong clinical recommendations can be made.

## Data Availability

The original contributions presented in the study are included in the article/Supplementary Material, further inquiries can be directed to the corresponding author/s.
